# Adipose Tissue Heterogeneity: Depot-Specific Location and Functional Specialization in Obesity-Related Disease

**DOI:** 10.3390/ijms27156693

**Published:** 2026-07-27

**Authors:** Mara Patricia Chávez-Ortega, Mario Peña-Peña, Roxana Carbó, Aida Medina-Urrutia, Horacio Osorio-Alonso, Baohong Jiang, Julio C. Almanza-Pérez, Gerardo Blancas-Flores, Santiago Villafaña, Rodrigo Romero-Nava, Fausto Sánchez-Muñoz, Fengyang Huang

**Affiliations:** 1 Posgrado en Biología Experimental, División de Ciencias Biológicas y de la Salud, Universidad Autónoma Metropolitana, Ave. San Rafael Atlixco 186, Mexico City 09340, Mexico; marachavezfth@gmail.com; 2 Laboratorio de Investigación en Obesidad y Asma, Hospital Infantil de México Federico Gómez, St. Dr. Márquez 162, Mexico City 06720, Mexico; 3 Departamento de Fisiología, Instituto Nacional de Cardiología Ignacio Chávez, Juan Badiano 1, Mexico City 14080, Mexico; marionutricion2017@gmail.com; 4 Departamento de Biomedicina Cardiovascular, Instituto Nacional de Cardiología Ignacio Chávez, Juan Badiano 1, Mexico City 14080, Mexico; roxcarbo@gmail.com; 5 Departamento de Endocrinología, Instituto Nacional de Cardiología Ignacio Chávez, Juan Badiano 1, Mexico City 14080, Mexico; aidaxm@yahoo.com; 6 Departamento de Fisiopatología Cardio-Renal, Instituto Nacional de Cardiología Ignacio Chávez, Juan Badiano 1, Mexico City 14080, Mexico; horace_33@yahoo.com.mx; 7 Shanghai Institute of Materia Medica, Chinese Academy of Sciences, 501# Haike Road, Shanghai 201203, China; jiangbh@simm.ac.cn; 8 Laboratorio de Farmacología, Departamento de Ciencias de la Salud, División de Ciencias Biológicas y de la Salud, Universidad Autónoma Metropolitana, Ave. San Rafael Atlixco 186, Mexico City 09340, Mexico; jcap@xanum.uam.mx (J.C.A.-P.); gera@xanum.uam.mx (G.B.-F.); 9 Laboratorio de Terapia Génica Experimental, Escuela Superior de Medicina, Instituto Politécnico Nacional, Sección de Estudios de Posgrado e Investigación, Mexico City 11340, Mexico; svillafana@ipn.mx; 10 Laboratorio de Investigación en Genética de Enfermedades Metabólicas, Escuela Superior de Medicina, Instituto Politécnico Nacional, Sección de Estudios de Posgrado e Investigación, Mexico City 11340, Mexico; roloromer@gmail.com

**Keywords:** adipose tissue, adipocyte, diversity, obesity, inflammation, insulin resistance, adipose tissue dysfunction

## Abstract

Adipose tissue is now recognized as a heterogeneous endocrine and metabolic organ rather than a passive lipid reservoir. Beyond the classical white, brown, and beige adipocytes, several depot- and organ-specific lipid-storing cell populations, including pink adipocytes, bone marrow (yellow) adipocytes, and hepatic stellate cells, contribute to energy homeostasis, thermogenesis, immune regulation, bone marrow function, and hepatic retinoid storage. Most existing reviews address these populations separately or focus narrowly on classical depots. Here, we integrate classical and non-classical adipocyte populations within a single depot-specific framework, examining how anatomical location and functional specialization jointly determine their contribution to obesity-related non-communicable diseases, including type 2 diabetes mellitus, metabolic-associated fatty liver disease, hypertension, and atherosclerotic cardiovascular disease. We further highlight unresolved mechanistic questions, including macrophage phenotypic heterogeneity beyond the classical M1/M2 model, the translational limits of brown adipose tissue activation, and the paracrine role of perivascular adipose tissue, that represent priority areas for future investigation.

## 1. Introduction

Obesity is defined as an excessive accumulation of adipose tissue that can impair biological functions [1] and is a complex health issue influenced by genetic, psychological, sociocultural, and economic factors [2]. In 2024, the World Atlas of Obesity indicated that the global prevalence of obesity has increased significantly, especially in low-income countries [3]. Consequently, the World Health Organization (WHO) is unlikely to achieve its 2025 targets for reducing obesity rates to 2010 levels [1,2,3]. In contrast, projections indicate that by 2035, 24% of the world’s population (approximately 1.77 billion people) will be classified as obese [3].

These epidemiological trends support the relevance of obesity as a major risk factor for several non-communicable diseases (NCDs) [4,5]. NCDs affect approximately 80% of individuals with obesity and are the leading cause of mortality worldwide [4]. Among the most critical NCDs associated with obesity are cardiovascular disease, metabolic-associated fatty liver disease (MAFLD), and type 2 diabetes mellitus (T2DM) [6,7].

A deeper understanding of the mechanisms linking adipose tissue expansion to obesity-related diseases is therefore essential for clarifying the biological basis of these complications [8,9,10]. Current evidence indicates that excessive adipose tissue accumulation in specific anatomical regions is particularly relevant, as adipocyte dysfunction within these depots may contribute to local and systemic metabolic alterations [11]. Different types of adipocytes as well as other lipid-storing specialized cells such as hepatic stellate cells are affected by the development of obesity. Thus, adipose tissue should not be interpreted solely as a pathological structure, but as a tissue whose adaptive or maladaptive responses depend on biological context [12].

The inclusion of non-classical, adipocyte-like populations alongside classical white, brown, and beige adipose tissue in this review is not intended to imply a shared developmental origin. Rather, these populations are grouped because they share three features directly relevant to obesity pathophysiology: intracellular lipid storage as a core structural and functional property, regulation that is specific to their anatomical or organ microenvironment (bone marrow, liver, breast, vasculature), and demonstrated plasticity or dysfunction in response to the obese state. Framing these diverse cell types within a single depot-specific model allows their shared and divergent contributions to metabolic disease to be compared directly, which is the central aim of this review.

For this reason, this review provides an overview of the role of adipose tissue in the progression of obesity-associated diseases, with particular attention to conditions affecting cardiovascular and metabolic health. This analysis also emphasizes how adipocyte anatomical location and morphology influence biological processes that are essential for tissue function and systemic homeostasis.

## 2. Adipose Tissue: A Journey of Discovery

Adipose tissue was long regarded as an inert structure providing mechanical support and thermal insulation, following early descriptions of lipid-laden polygonal cells in 1846 [13]. This view was progressively overturned across the 20th century: studies in the following decades showed that lipid droplet size responded to feeding state [14], that adipose tissue participated in energy-homeostatic signaling from nerves and hormones [15,16,17,18], and that morphologically distinct adipocyte populations existed within the tissue, later identified as brown adipocytes [19,20,21].

A pivotal conceptual shift occurred with the discovery that adipocytes secrete signaling molecules, adipokines, beginning with adipsin and followed by leptin and adiponectin [22,23,24,25], establishing adipose tissue as a bona fide endocrine organ now known to produce more than 60 such factors with systemic effects on the brain, liver, pancreas, cardiovascular system, and immune system [26,27]. In parallel, epidemiological studies in the 1980s linked fat accumulation in specific anatomical regions, rather than total adiposity, to the risk of type 2 diabetes, hypertension, and cardiovascular disease [28,29,30,31,32], establishing depot location as a determinant of metabolic risk.

Collectively, these discoveries transformed adipose tissue from a presumed passive depot into a heterogeneous, secretory, and anatomically diverse organ whose dysfunction actively drives systemic disease. This reconceptualization underlies the depot-specific framework adopted throughout the present review: rather than treating adipose tissue as a single functional entity, we examine how location-specific and lineage-specific differences between adipocyte populations translate into distinct contributions to obesity-related pathology.

## 3. Physiology of Adipose Tissue, Adipocytes and Its Role in Obesity-Related Disease Progression

**Figure 1:**
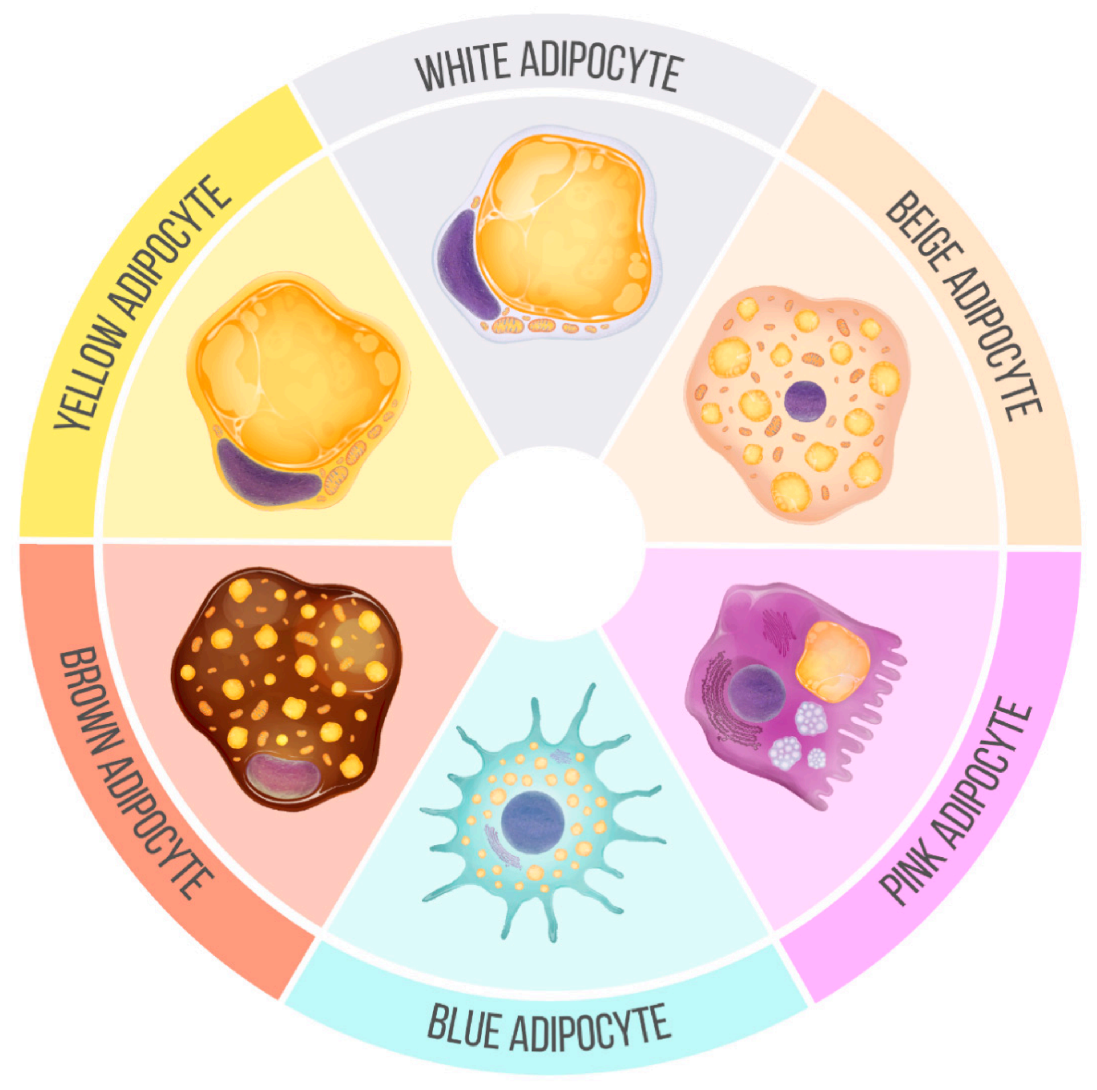
Morphology of adipocytes: white, brown, beige, pink (breast tissue), yellow (bone marrow), and blue adipocyte (hepatic stellate cells).

In healthy individuals, adipose tissue accounts for approximately 20–30% of total body weight and is mainly composed of triglycerides stored within adipocytes [33]. As previously mentioned, adipocytes are the main cellular component of adipose tissue. They are characterized by an oval shape, variable diameter, one or more lipid droplets, thin cytoplasm, and a nucleus that is displaced toward the periphery [34,35]. Adipocytes constitute approximately 60–70% of adipose tissue cells; however, this tissue also contains a stromal vascular fraction (SVF) embedded within an extracellular matrix [35,36]. SVF includes capillaries, endothelial cells, nerves, adipocyte precursor cells, and immune cells, including macrophages, T lymphocytes, and mast cells [36]. In humans, adipose tissue begins to form during intrauterine development and, in most individuals, continues to remodel throughout life [37,38]. This morphological and functional diversity supports classifying adipose tissue into several types, as summarized in Figure 1.

### 3.1. Developmental and Embryological Origins of Adipocyte Populations

Adipocyte populations arise from distinct embryonic precursor lineages, which partly explain their functional divergence. Classical brown adipocytes derive from Myf5-expressing precursors in the dermomyotome, a lineage shared with skeletal myocytes, consistent with the shared mitochondrial and oxidative machinery between brown fat and muscle [39]. White adipocytes were originally thought to arise exclusively from Myf5-negative precursors; however, lineage-tracing studies show that a subset of white adipocytes in some subcutaneous depots also derives from Myf5-lineage cells, and that visceral adipocytes may arise from a distinct Wt1+ lateral-plate-mesoderm precursor population separate from subcutaneous precursors [40]. More recent single-cell RNA sequencing of human subcutaneous adipose tissue confirms that precursor populations differ by depot even within subcutaneous fat, since abdominal versus gluteofemoral precursors show distinct commitment and metabolic gene signatures, indicating that depot-specific developmental programming persists into adult human tissue and is not simply a rodent phenomenon [41]. Beige adipocytes, interspersed within white adipose tissue and induced by cold or β3-adrenergic stimulation, do not arise from the Myf5 lineage; their precursor identity remains incompletely defined. Furthermore, the precursor identity of beige adipocytes and the extent to which rodent lineage-tracing models generalize to humans remain open questions requiring further human-tissue lineage studies.

### 3.2. White Adipose Tissue (WAT) and White Adipocytes

White adipose tissue (WAT) is the most extensively studied adipose depot, partly because it is present throughout life and represents the main site of lipid storage [42]. It is also the most abundant, accounting for approximately 10–20% of body weight in an adult individual [43]. Its pale appearance is related to its moderate vascularization and the predominance of lipid-loaded adipocytes [42]. These adipocytes are characterized by a large unilocular lipid droplet, which supports efficient energy storage [42,43]. The formation of adipocytes during intrauterine growth begins with undifferentiated mesenchymal cells forming lipoblasts at the periphery of blood vessels through the activation/repression of transcription factors [38]. With the appearance of slight lipid inclusions near the nucleus, lipoblasts acquire an oval shape and are called intermediate lipoblasts [35]. When lipid droplets coalesce into a large droplet, the cells are termed mature adipocytes [38,42].

Mature white adipocytes, which vary in size from 20 to 200 micrometers in diameter, demonstrate remarkable structural plasticity [38]. This variability enables adipocytes to expand or contract in response to an individual’s physiological state, sex, and tissue location [38,42]. WAT can expand through two mechanisms: hyperplasia, an increase in the number of adipocytes, and hypertrophy, an increase in adipocyte size [38,42,43]. Both mechanisms are influenced by age. During early life, WAT expansion occurs through both hyperplasia and hypertrophy [44]. However, tissue hyperplasia gradually decreases during adulthood, and hypertrophy predominates [44,45].

WAT hyperplasia is a process modulated by insulin, insulin-like growth factor 1 (IGF-1), and fibroblast growth factor 1 (FGF-1) [45]. In the case of hypertrophy, the peroxisome proliferator-activated receptor gamma (PPAR-γ) plays a crucial role by regulating fatty acid storage and suppressing lipolysis [44,45]. The presence of modulators of these WAT expansion mechanisms is crucial because the number and size of adipocytes are key factors in metabolic health [43,45]. A reduction in adipogenic capacity, together with an increase in white adipocyte size, is associated with WAT dysfunction [43].

Before considering its role in disease, it is important to note that healthy WAT performs several essential physiological functions beyond lipid storage. As an endocrine organ, WAT secretes adiponectin, leptin, and other adipokines under basal, non-inflammatory conditions to regulate systemic insulin sensitivity, food intake, and energy expenditure [26,27]. Its lipid buffering capacity, the ability to expand adipocyte number and size in response to caloric surplus, protects non-adipose tissues (liver, muscle, pancreas) from ectopic lipid accumulation, provided this expansion occurs primarily through hyperplasia rather than hypertrophy [43,44,45]. Healthy WAT also demonstrates metabolic flexibility, shifting between lipogenesis and lipolysis according to feeding state and energy demand, coordinated by insulin and catecholamine signaling. Loss of this flexibility, rather than adiposity per se, is increasingly recognized as a determinant of metabolic dysfunction, underscoring that WAT pathology reflects a failure of adaptive capacity rather than an intrinsic property of the tissue itself.

**Figure 2:**
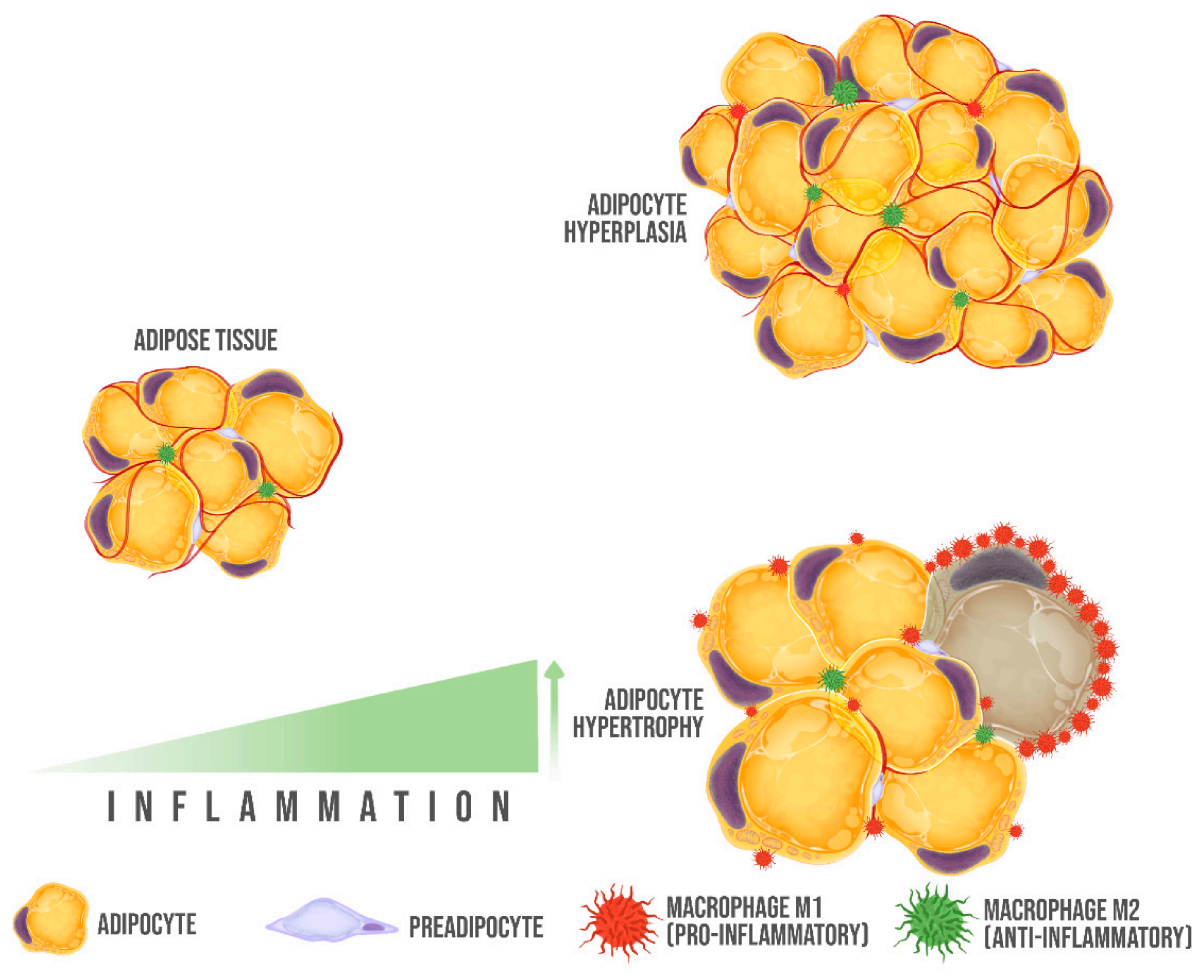
Expansion of adipose tissue through hyperplasia (an increase in the number of adipocytes) and hypertrophy (an increase in the size of adipocytes), and its association with the progression of obesity. The green gradient and upward arrow indicate increasing inflammation, whereas the gray-brown adipocyte represents a hypertrophic adipocyte.

When white adipocytes become hypertrophic, they may initiate coordinated changes in the tissue microenvironment that lead to structural remodeling (Figure 2) [38,43]. Recently, spatially resolved single-nucleus analyses of human abdominal subcutaneous adipose tissue showed that obesity is associated with senescence-prone metabolic, precursor, and vascular cell states. In contrast, weight loss reverses much of this remodeling but does not completely normalize macrophage activation. Hypertrophic WAT alters its secretory profile, increasing the release of inflammatory mediators (visfatin, interleukin-6, interleukin-8, tumor necrosis factor alpha, and resistin) and decreasing the secretion of adipocytokines with anti-inflammatory actions (interleukin-10, interleukin-4, and interleukin-13) [46]. It also increases leptin release, which regulates food intake in the hypothalamus, and conversely decreases adiponectin secretion, thereby reducing insulin sensitivity, lipid oxidation, and modulation of inflammation [43,46,47]. Furthermore, hypertrophic WAT promotes the infiltration and activation of macrophages, which are classified into two main subtypes: M1, with pro-inflammatory properties, and M2, with an anti-inflammatory profile [42]. Although this classification has been well described in experimental models, its relevance and delineation in humans are less specific [48,49]. Hypertrophic WAT exhibits an activated inflammatory M1 phenotype, comprising up to 40% of WAT cells.

However, single-cell genomic studies have identified additional adipose tissue macrophage (ATM) subpopulations that do not fit this binary, including metabolically activated macrophages (MMe), lipid-associated macrophages (LAMs) and their precursor state (pre-LAM), Mox macrophages, and perivascular macrophages (PVMs) that decline in number with obesity [50]. A 2024 single-cell genomics review notes that although several ATM clusters expand with obesity, none of them truly resembles the classical M1 phenotype described in early rodent work, underscoring that the field has moved well past the binary model [51]. But research is lacking on whether specific intermediate ATM phenotypes are causally linked to insulin resistance in humans, or are secondary markers of tissue remodeling.

Other consequences of sustained hypertrophy include extracellular matrix accumulation and insufficient angiogenic adaptation, which may contribute to fibrosis and WAT hypoxia [43]. Hypoxia, in turn, inhibits white adipocyte hyperplasia, generates local and systemic inflammation, promotes apoptosis in WAT, and leads to insulin resistance (IR) [38,42,43,44,46].

The implications of IR are significant, given that insulin plays a crucial role in regulating glucose and lipid metabolism within white adipocytes [49]. In healthy individuals, insulin exerts its effect by binding to its receptor, which autophosphorylates on tyrosine residues and phosphorylates the insulin receptor substrate 1 (IRS-1) [49,52]. The activation of IRS-1 facilitates the formation of molecular complexes within the phosphatidylinositol-3-kinase (PI3K) pathway. Consequently, glucose can enter the adipocyte through the translocation of glucose transporter 4 (GLUT-4) [49]. In contrast, in individuals with IR, the hormone’s ability to perform its biological functions is diminished [53]. This alteration is mainly due to reduced insulin interaction with the receptor, its expression, and kinase activity [49,53]. Likewise, ISR-1 increases its phosphorylation at serine/threonine residues, altering its ability to associate and causing attenuation of GLUT-4 translocation [53].

Beyond IRS-1 serine/threonine phosphorylation, several converging pathways link adipocyte hypertrophy and inflammation to insulin resistance. Pro-inflammatory cytokines (TNF-α, IL-1β) activate c-Jun N-terminal kinase (JNK), which directly phosphorylates IRS-1 at inhibitory serine residues, and the IKKβ/NF-κB pathway, which impairs insulin signaling primarily through transcriptional upregulation of inflammatory mediators rather than direct IRS-1 modification [54,55]. Chronic nutrient overload also induces endoplasmic reticulum (ER) stress; the PERK-eIF2α and IRE1 branches of the unfolded protein response converge on both NF-κB activation and induction of the TXNIP-NLRP3 inflammasome, driving IL-1β secretion and further impairing insulin signaling [56]. Adipocyte mitochondrial dysfunction, characterized by increased reactive oxygen species production and impaired oxidative capacity, contributes to and is exacerbated by these inflammatory pathways, creating a feed-forward cycle. Together, JNK, IKKβ/NF-κB, ER stress, mitochondrial dysfunction, and inflammasome activation represent overlapping rather than independent mechanisms, converging on impaired IRS-1 function and reduced GLUT-4 translocation. The relative contribution of each pathway in human versus rodent adipose tissue, and whether they can be therapeutically targeted selectively, remains incompletely defined.

IR is a key factor in the development of T2DM and metabolic syndrome; however, when combined with WAT dysfunction, its impact extends beyond these conditions. IR reduces adipocytes’ ability to store lipids, increasing free fatty acid (FFA) concentrations [45,47]. Elevated circulating FFAs are associated with ectopic lipid accumulation, a process involved in the progression of several obesity-associated diseases [43,46,47].

For instance, the deposition of WAT in the liver and IR leads to ectopic fat accumulation in organs such as the liver, muscle, kidney, and pancreas. This promotes oxidative stress and mitochondrial dysfunction. Consequently, liver injury, inflammation, and fibrosis, which are hallmarks of MAFLD, are promoted [52]. Additionally, for the development of cardiovascular diseases, atherosclerosis, ventricular hypertrophy, and diastolic dysfunction, the accumulation of pro-inflammatory macrophages, hypertriglyceridemia, epicardial WAT deposition, and inflammation play a crucial role [57]. In hypertension, it is essential to consider that insulin plays a role in blood pressure regulation [52,58,59], since it promotes vasorelaxation by stimulating nitric oxide production in the endothelium and modulates sodium reabsorption in the kidney [59]. Consequently, 80% of patients with T2DM also have hypertension [59,60]. Moreover, certain types of cancer are frequently associated with WAT inflammation, as the accumulation of macrophages, hypoxia, and increased cytokine secretion promote the activation of pathways involved in carcinogenesis [61], for example, tumor necrosis factor-alpha (TNF-α), which in certain circumstances can be a tumor promoter by activating cell life/death signaling pathways such as the nuclear factor kappa B (NF-κB) pathway and specific mitogen-activated protein kinase (MAPK) cascades involved in apoptotic or survival processes [52,57,61]. Finally, cognitive decline is also a consequence of IR. Therefore, in obese middle-aged patients with glucose intolerance, there is a risk of developing dementia, memory loss, or even neurodegenerative diseases such as Alzheimer’s increases [62,63]. Likewise, recent studies indicate the association between increased secretion of adipokines such as visfatin and cystatin C with the development of psychiatric disorders, multiple sclerosis, and Alzheimer’s [63].

Considering the association between WAT and the obesity comorbidities mentioned above, WAT has been extensively studied for many decades. On the one hand, in vivo, models have been implemented in animals induced to obesity through mutations in leptin (ob/ob mouse, Zucker rat, etc.), by changes in diet (high-fat diet, high-carbohydrate diet, etc.), or by surgical and chemical methods (ovariectomy, lesion of the ventromedial hypothalamus or the hypothalamic paraventricular nucleus) [64,65,66,67].

WAT is identified by the expression of genes such as Hoxc8 and Hoxc9, which exhibit region-specific patterns [68]. Hoxc8 is predominantly expressed in intra-abdominal adipose depots, such as epididymal and perirenal fat, whereas Hoxc9 is more associated with subcutaneous fat depots [69]. However, neither gene is an exclusive marker of adipocytes, as their expression extends to other cellular components within the stromal vascular fraction of adipose tissue [68,69]. However, the amino acid transporter ASC-1 is a white adipocyte-specific cell surface protein with low expression in other adipocytes [68].

These genes are part of broader transcriptomic signatures that vary by adipose depot and developmental stage, reflecting the complex biology of adipose tissue rather than serving as definitive markers for WAT [69,70]. In the case of in vitro and ex vivo models, mouse cell lines (3T3-L1, 3T3-F442A, C3H/10T1/2) or human cell lines (immortalized cell lines, human adipose tissue-derived stem cells) are implemented [70]. To corroborate the presence of white adipocytes, some commonly used surface markers are Serpina3K, Wdnm1-like, and Asc1 [68].

As a result of extensive research, various strategies have been developed to control excessive WAT growth and manage obesity. Traditional approaches, such as dietary therapy, focus on reducing lipid intake, portion sizes, and energy content while incorporating fruits and vegetables [71,72,73]. Physical activity and regular monitoring by health professionals remain integral components of long-term weight management [71,74]. Pharmacological treatments have undergone significant evolution over the years. Incretin mimetics, such as GLP-1 receptor agonists (e.g., semaglutide and liraglutide), have emerged as effective therapies for obesity and related metabolic disorders [75]. These agents promote weight loss by enhancing satiety, delaying gastric emptying, and improving insulin sensitivity. Similarly, melanocortin-4 receptor (MC4R) agonists, such as set-melanotide, represent a breakthrough for rare genetic forms of obesity by regulating appetite and energy homeostasis [76]. Older pharmacological options like phentermine, benzphetamine, and orlistat, while still in use, have been complemented by these novel agents [67,74]. Bariatric surgery remains a highly effective option for individuals with a BMI ≥ 40 kg/m^2^, resulting in significant and sustainable weight loss, as well as reduced prevalence of DM, dyslipidemia, and hypertension [77,78].

Dietary supplements have also been explored as complementary strategies for weight management and metabolic modulation [79,80]. Some commonly used products include spirulina, chitosan, green tea extract, probiotics, and omega-3 polyunsaturated fatty acids (ω-3 PUFAs) [79]. The latter is mainly composed of eicosapentaenoic acid (EPA) and docosahexaenoic acid (DHA) [79,81]. So far, ω-3 PUFAs appear to have therapeutic potential for heart disease, T2DM, and MAFLD due to their anti-inflammatory, hypolipidemic, and insulin-sensitizing effects [81,82,83]. It is essential to note that, despite the variety of supplements and evidence of their health benefits, the long-term effectiveness of these products remains to be determined [79,81].

### 3.3. Brown Adipose Tissue (BAT)

Brown adipose tissue (BAT) also stores lipids, as in WAT; however, in humans, BAT is much less abundant and rarely exceeds 1% of body weight [84]. Moreover, the morphology, origin, and function of its adipocytes differ. BAT adipocytes are characterized by their small size (10 to 25 micrometers in diameter), storing triglycerides in multiple lipid droplets (multilocular), having a rounded nucleus, and a reduced endoplasmic reticulum [34,35]. The distinctive color of BAT reflects its dense vascularization, rich sympathetic innervation, and high mitochondrial content [36,42,85,86,87]. Regarding their origin, brown adipocytes form during embryonic development, as do white adipocytes. However, BAT adipocytes share the same lineage as skeletal muscle myocytes [42,85,86].

Unlike WAT, BAT’s primary function is not energy storage. On the contrary, it specializes in lipid catabolism and adaptive thermogenesis [42]. This function is conferred by the uncoupling protein 1 (UCP-1) located in the inner membrane of its mitochondria [83,84,88,89]. UCP-1 uncouples oxidative phosphorylation, dissipating the proton electrochemical gradient across the mitochondrial membrane that is typically used to generate ATP [86,87,88]. As a result of this mechanism, energy is dissipated as heat, and fatty acid oxidation is stimulated [88,89]. UCP-1 activation is triggered by exposure of the organism to cold, fatty acids, the release of insulin, and norepinephrine by the sympathetic nervous system [90,91].

Due to its primary function of thermogenesis, BAT was initially thought to be limited to hibernating mammals and was therefore referred to as the hibernation gland [82]. However, it was later discovered that this tissue is also present in other non-hibernating mammals, such as rats, mice, cats, and humans [86,88]. BAT is now recognized as essential for thermoregulation and survival in newborn small mammals. Since they are predisposed to lose heat due to their body volume-to-surface area ratio [86,88], they lack sufficient muscle mass to regulate their temperature through shivering [86].

**Figure 3:**
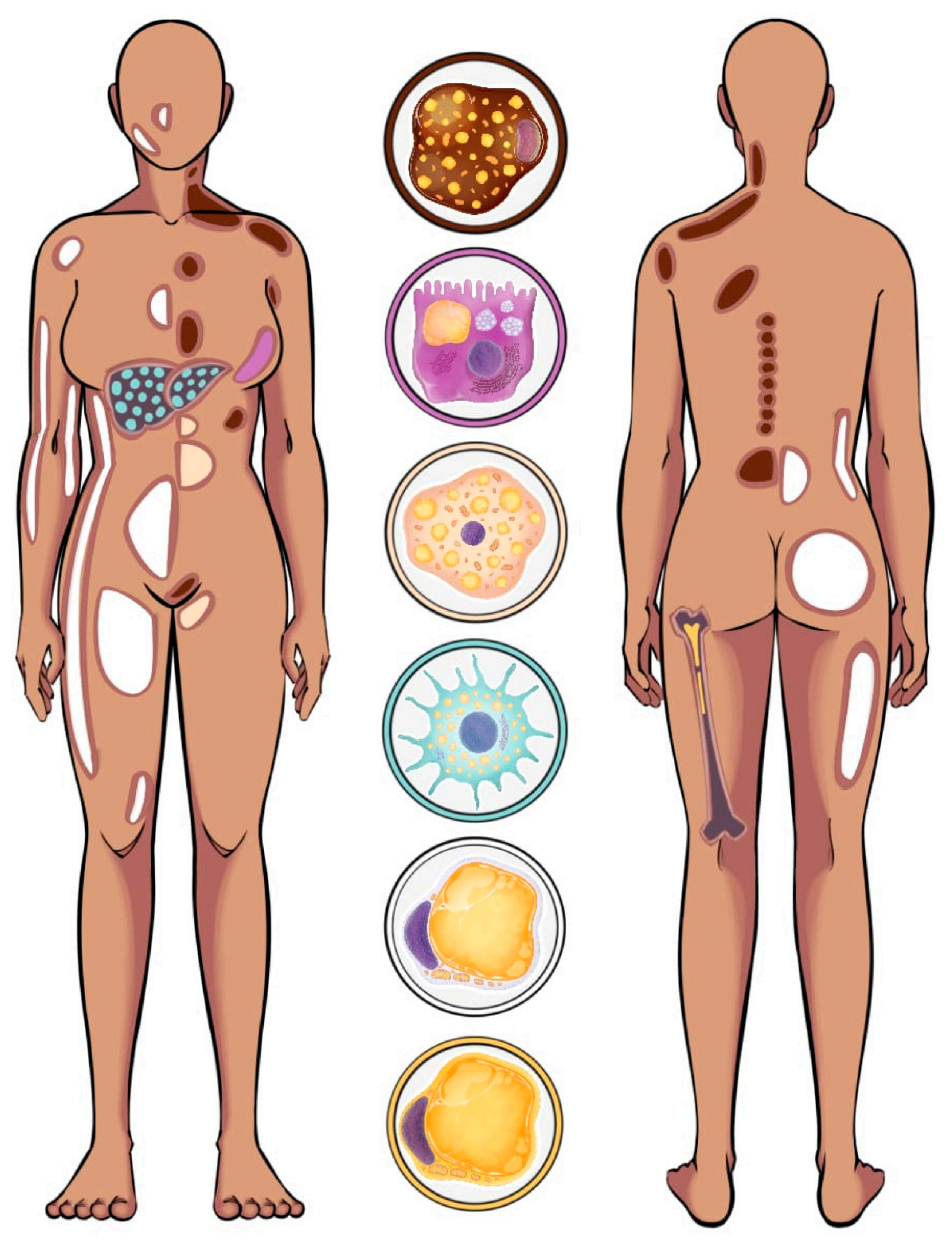
Anatomical location of adipose tissue: white, brown, beige, pink (mammary tissue), yellow (bone marrow), and blue (hepatic stellate cells).

Regarding its location, BAT in rodents accumulates in the interscapular, subscapular, perirenal, and periaortic regions [85]. In human neonates, BAT is primarily distributed in the interscapular, supraclavicular, and paravertebral regions [86,87]. Finally, studies on adult humans have been limited until recently, as it was previously thought to be present only in newborns. In 2007, evidence indicated that BAT does not completely atrophy during childhood [92]. On the contrary, a portion of BAT is preserved in the supraclavicular and paravertebral areas (Figure 3) [92,93]. However, BAT in adults differs from that previously described in small mammals, as adipocytes contain less UCP-1 and are surrounded by white adipocytes [94,95]. Evidence supports the existence of metabolically active BAT in adults, which regulates total body energy expenditure and body fat. Additionally, BAT presence is influenced by diabetic status or blood glucose levels [96].

In contrast to WAT, BAT does not appear to increase with obesity, and studies even indicate a negative correlation between BAT activation and body mass index (BMI) [83,86]. Furthermore, BAT is notably reduced in individuals with T2DM [91,94]. The mechanism by which BAT decreases is not well defined; however, it has been suggested that it may result from insulin resistance [94,97]. For this reason, BAT activation is generally not considered a driver of obesity-related comorbidities. Accordingly, BAT activation has been explored as a potential therapeutic strategy for conditions associated with excessive WAT expansion [84,93,94]. BAT is a tissue capable of increasing energy expenditure through thermogenesis using substrates such as triglycerides (primarily), glucose, and some amino acids [42].

Cold exposure is considered the classical stimulus for BAT activation [90,91]. This mechanism occurs in brown adipocytes via a signal transduction pathway initiated by norepinephrine binding to the β3-adrenergic receptor (β3-AR) [90,98,99]. This signaling pathway promotes UCP-1 expression and lipid β-oxidation to produce heat [98]. Currently, studies in rodents exposed to intense cold (between 4 °C and 10 °C) for extended periods show that brown adipocyte activity increases by approximately 1.5-fold [100,101]. In related human studies, daily exposure to cold at 17 °C for 2 h over 6 weeks has been shown to significantly increase BAT activity and reduce body fat mass [101]. However, a limitation is that this cold exposure is not readily applicable to humans [100]. Similarly, systemic increases in norepinephrine are not selective and may adversely affect cardiovascular function and cognition [84,100]. For this reason, researchers are currently seeking to develop selective methods of BAT activation [80], for example, supplements (capsaicin, ephedrine, catechin, and retinoic acid) or BAT transplantation [84,91,99].

So far, some positive effects of BAT activation include reductions in hyperglycemia and dyslipidemia typical of T2DM and metabolic syndrome [97,102]. It is suggested that 40 g of active BAT could promote approximately 20% of the total energy expenditure of an adult [102]. In cardiovascular diseases, experiments in rodents have indicated that BAT has a protective effect [98,102]. In addition to its thermoregulatory function, this tissue is also a secretory organ. Some adipokines secreted by BAT, such as fibroblast growth factor 21 (FGF21), delay the development of hypertension and protect the heart from inflammation, oxidative stress, and decreased HDL [102,103]. Likewise, in mice with hyperlipidemia, the administration of β-adrenalin (to promote BAT activation) delayed the development of atherosclerosis [102]. This effect is associated with the increased expression of heme oxygenase, mediated by adipocyte-specific overexpression of the peroxisome proliferator-activated receptor gamma coactivator 1-alpha (PGC-1α), which contributes to vasodilation and improved vascular function [102,103]. Regarding cancer, recent studies in mice suggest that BAT activation could reduce the size of fibrosarcoma, melanoma, and pancreatic cancer tumors [104]. To date, it is suggested that the decrease in glucose supply to the tumor microenvironment could decrease hypoxia, angiogenesis, and cell proliferation [104,105].

Despite the growing interest in BAT for its therapeutic potential, its study, even to date, remains a challenge. Some techniques used to identify brown adipocytes based on their morphology are light microscopy, transmission, and scanning electron microscopy [106,107]. These approaches allow the identification of multilocular adipocytes with high mitochondrial density and abundant cristae [106]. Primary cell cultures and BAT explants are also implemented [108]. These are proliferated and differentiated in vitro, with the SV40 T antigen used for immortalization. Identification is achieved by analyzing BAT-specific markers such as UCP-1, Cidea, and Prdm16, although these are not surface markers but intracellular or transcriptional markers [68,108,109]. Finally, imaging techniques such as magnetic resonance imaging are applied for the non-invasive study of the structure and function of BAT in vivo (animals and humans) [110]. However, despite being a cost-effective method, it is not entirely accurate because it is based on the water and fat content of BAT [110,111]. For this reason, for some years now, the preferred method for characterizing BAT has been positron emission tomography with fluorine-18-fluorodeoxyglucose (18 FDG-PET/CT) [111,112]. However, it is warned that this technique also has limitations. For example, 18 FDG-PET/CT determines the absorption of glucose in BAT, which does not accurately reflect its thermogenic activity since triglycerides are its primary energy source [111].

Despite these promising findings, several translational barriers temper the therapeutic outlook for BAT activation. Metabolically active BAT mass in adult humans is limited, constraining its maximal contribution to whole-body energy expenditure regardless of activation strategy [113]. BAT presence and activity also show marked interindividual variability, influenced by age, sex, adiposity, and cold-exposure history [114]. The durability of pharmacologically or cold-induced BAT activation is also uncertain. Together, these findings suggest that human BAT, even under optimized activation, may be insufficient on its own to meaningfully reverse obesity-associated cardiometabolic disease, and is more likely to require combination with other interventions rather than deployment as a standalone therapy [114]. It is worth noting that selective pharmacological BAT activators without systemic adrenergic side effects, and reliable methods to quantify thermogenic (not just glucose-avid) BAT activity in vivo, remain unmet needs. Therefore, several opportunities remain to further investigate BAT function. More specific methods for studying BAT are needed to clarify the therapeutic relevance of BAT activation in individuals with obesity and metabolic dysfunction.

### 3.4. Beige Adipose Tissue (BeAT)

In addition to BAT, another type of thermogenic adipose tissue called beige or brite adipose tissue (BeAT) was initially described in 2008 by Seale and colleagues [115]. Because BeAT is present in limited and heterogeneous depots in humans, its total proportion has not been precisely established, although it appears to be comparable to that of BAT [116,117]. BeAT shares a mesenchymal origin with WAT and is found within subcutaneous cervical, supraclavicular, axillary, and inguinal depots, where beige adipocytes are interspersed among white adipocytes (Figure 3) [110,116,117]. Although its localization is heterogeneous, beige adipocyte morphology resembles that of brown adipocytes, with centrally located nuclei, multilocular lipid droplets, and abundant mitochondria [117].

The primary function of BeAT is thermoregulation, and beige adipocytes are often considered inducible or transient thermogenic cells. However, BeAT can also store energy because it exhibits an intermediate phenotype: it may express UCP-1, as observed in BAT, or store lipids in droplets, as occurs in WAT [117,118,119]. The mechanism through which this occurs is called browning, and even today, it is not fully understood. To date, two models have been proposed: the first suggests that WAT has the property of converting to BeAT through a reversible transdifferentiation process; the second indicates that BeAT adipocytes come from multiple precursor lineages that, when active, share characteristics with BAT and, when inactive, resemble WAT [116,120]. Despite this uncertainty, we currently know that, upon the activation of BAT, browning occurs in response to prolonged exposure to cold and β-adrenergic stimulation [37,86,117,120,121].

Recently, Wang et al. described that two distinct subpopulations of beige adipocytes coexist to dissipate heat. Heat dissipation may occur through UCP-1-dependent mechanisms or ATP-dependent futile cycles (FCs), defining UCP-1 beige adipocytes and FC adipocytes, respectively. The FC adipocyte subpopulation is highly metabolically active and uses substrate cycles involving creatine, calcium, and lipids, resulting in net ATP consumption and UCP-1 independent energy dissipation [122]. These cells are essential to direct systemic energy homeostasis and are linked to glucose metabolism and obesity resistance in humans and regulators of energy homeostasis in mammals.

Studies indicate that, since active BeAT increases insulin sensitivity and can catabolize lipids (like BAT), it does not increase in obesity (like WAT) [110,120,121]. However, recent studies indicate that, in the presence of inflammation in BeAT adipocytes, the expression of the UCP-1 protein decreases, and its predominant function is lipid storage [121,123,124]. For this reason, BeAT activation and WAT browning have attracted interest as potential strategies for metabolic disorders associated with obesity [121].

As previously mentioned, the primary stimulus for BeAT browning is cold; however, in recent decades, other alternatives have been explored to promote the activity of this tissue [117,120,121]. For example, exercise increases irisin secretion in skeletal muscle, promoting white adipocyte browning [120,121,125,126]. Therefore, in animal models, it increases energy consumption, reduces body weight, decreases WAT, and improves glucose tolerance [125,126]. Studies have indicated that these effects were potentiated by implementing a balanced diet [125]. Likewise, the intervention of dietary components such as resveratrol, curcumin, and omega-3 polyunsaturated fatty acids (EPA and DHA) also promotes the browning of WAT and thermogenesis in obese rodents [127,128,129,130]. To date, it is suggested that this effect results from activating the AMPK/SIRT1/PGC-1α pathway and the transcription factors PPAR-α/γ [117,119,120,127].

However, it is essential to note that research on the BeAT presents a challenge due to its physiological and morphological similarities to BAT [37,121]. Primary cell cultures (3T3-L1 preadipocytes subsequently differentiated) or BeAT explants are identified with surface markers such as Tmem26, Tbx1, and CD137 [37,131]. In addition, the amino acid transporter PAT2 and the purinergic receptor P2RX5 are cell surface markers expressed in brown and beige adipocytes in mice. These markers also selectively mark brown/beige and white adipocytes in human tissue [68].

Regarding in vivo studies, techniques such as the Bruker In Vivo Xtreme imaging system or multispectral optoacoustic tomography are commonly used to detect BeAT in rodents due to being minimally invasive [131]. Finally, radiopharmaceuticals such as 18 FDG-PET/CT and TPSO-18 kDa are the most optimal imaging modalities [131,132,133]. It is important to note that to understand further what we know about BeAT and the extent of the activation of these adipocytes on the metabolic state, it is essential to find better BeAT markers and more optimal techniques capable of distinguishing BeAT from BAT [37,126].

### 3.5. Pink Adipose Tissue (PAT)

Pink adipose tissue (PAT) was characterized in murine models over the past two decades [134]. Since its initial mention, its essential participation in the alveolar epithelium of the mammary glands during milk production and secretion has been established [134,135,136]. Additionally, it was indicated that pink adipocytes, like white, brown, and beige adipocytes, can store lipids [136]. However, their morphology differs, comprising brick-like epithelial cells [134,137]. PAT adipocytes are equipped with multiple lipid droplets that contain milk protein inside and are bigger than those observed in brown adipocytes [134,138,139]. Additionally, pink adipocytes have a thin cytoplasm, a centrally located nucleus, a developed Golgi apparatus, abundant rough endoplasmic reticulum (RER) cisternae, and larger mitochondria than those present in white adipocytes [134,139]. Regarding its origin, PAT comes from the subcutaneous adipose tissue of the mammary gland [134,138], which, through physiological stimuli during pregnancy, undergoes a transdifferentiation process commonly called “pinking” [134,139].

Before pregnancy, the mammary glands of females comprise a large amount of white adipose tissue infiltrated in branched ducts that end in the nipple [134,136]. However, during pregnancy and lactation, a milk-producing structure is formed, integrated by adipose epithelial cells (pink adipocytes) that supply energy to newborns through feeding (Figure 3) [134,136]. This event is called alveologenesis and occurs in females from the first 18 days of pregnancy [134,138]. The process begins when the alveoli of the mammary glands expand progressively until they occupy 90% of the mammary volume [135]. These milk-producing and secreting structures persist until the end of lactation and present a pink color at the macroscopic level, giving their characteristic name to PAT [134,136,140]. After lactation, the mammary gland begins an involution process during the first days [134]. These initiate the progressive decrease of the alveolar structures until they are replaced by white adipose tissue, reverting to the anatomy of the breast before pregnancy [136,140]. In the case of PAT adipocytes, the number of adipo-epithelial cells decreases until they disappear, and in parallel, the presence of cells with the typical morphology of WAT is reincorporated [134,136].

It should be noted that the molecular mechanisms that allow the formation of pink adipocytes are not fully understood. However, pregnancy hormones and the paracrine activity of the mammary glands are involved [131]. Additionally, studies published in 2014 indicated that secreted phosphoprotein 1 (SPP1) plays a vital role in WAT transdifferentiation to PAT [140,141]. Likewise, for the transdifferentiation of PAT to WAT, the transcription factor PPAR-γ is key in the involution of the mammary gland [138,141].

Studies on PAT have contributed to the understanding of adipose tissue plasticity and suggest that transdifferentiation may represent a physiological property of the adipose organ [136,138]. This concept is supported by evidence that adipocyte conversion is not limited to the white to pink transition and its reversal [136]. Some studies with electron microscopy and lineage tracing indicate that PAT can also convert to BAT, although there is currently no evidence of BAT conversion to PAT [136,138].

It is important to note that, to date, a direct association between the formation of PAT and the development of metabolic diseases associated with obesity has not been established. Conversely, breastfeeding has been associated with reduced offspring susceptibility to obesity, cardiovascular disease, and T2DM [142,143]. Studies suggest that this is because pink adipocytes, in addition to milk, secrete hormones such as leptin and adiponectin [134,137,138,139].

In addition, interesting results in obesity-induced rodents concluded that the transdifferentiation of pink adipocytes decreased considerably during pregnancy [134,136]. However, the mechanisms by which increased WAT may impair adequate PAT formation remain unclear [144,145]. As for other pathologies associated with obesity, studies indicate that the partial involution of the mammary gland at the end of lactation results in developing a microenvironment rich in pro-inflammatory mediators that can lead to tumorigenesis [141,142,143,144,145]. Likewise, mammary adipose tissue secretes growth factors and an energy source that cancer cells can use for survival [141]. This explains why the risk of developing breast cancer increases transiently with pregnancy, and the incidence of death associated with this type of cancer increases within the first five years after childbirth [145,146].

To date, the study of this type of adipose tissue still involves multiple difficulties due to its technical limitations [134]. Histological studies, lineage tracing experiments, and PAT explants in rodents use the whey acidic protein (WAP) as a marker for identifying PAT adipo-epithelial cells [134,136,138,139]. Because PAT has been described relatively recently, it remains unclear whether WAT transdifferentiation into milk-producing structures occurs similarly in humans during pregnancy [137]. Likewise, much remains to be explored about the participation of these adipocytes in energy homeostasis in mothers and offspring [143]. Finally, the effect of obesity-related inflammation on PAT function remains to be elucidated [147].

### 3.6. Yellow Adipose Tissue (YAT)

Yellow adipose tissue (YAT) was initially considered inert and was thought to primarily fill bony spaces; however, it is now recognized as an active component of the bone marrow microenvironment (Figure 3). In terms of its morphology, the yellow adipocyte is comparable to the white adipocyte, as it has a lipid droplet (90% of its cell volume), a thin cytoplasm, a nucleus located at the periphery, and a moderate number of mitochondria [148,149,150]. Regarding size, yellow adipocytes are larger than brown and beige adipocytes, as it has a diameter between 40 and 90 micrometers [148,149].

Despite its morphological similarities with WAT, YAT has distinct function, including regulation of the bone marrow microenvironment and participation in hematopoiesis [149,151,152,153]. Additionally, evidence indicates that the triglycerides stored in YAT do not provide energy during periods of caloric restriction; however, this energy can contribute to bone regeneration [149,150]. Recent evidence indicates that YAT functions as an active endocrine compartment that participates in the regulation of bone metabolism (and probably systemic metabolism), as it is a secretor of adipokines such as adiponectin, leptin, and pro-inflammatory cytokines [149,151,154,155].

In the case of its origin, yellow adipocyte (like WAT) formation occurs from mesenchymal progenitor cells and with the regulation of PPAR-γ [143,156,157]. This age-dependent process occurs from the distal to the proximal area [149,155]. For this reason, yellow adipocytes are formed during childhood in the phalanges and, later in puberty, accumulate in the appendicular and axial skeleton (mainly in the trabecular cavity) [149,151,156]. In adults between 25 and 50 years old, bone marrow is composed of 70% YAT, making it integrate approximately 10% of the total fatty tissue in healthy adults [148,155,156]. However, men generally have a greater number of yellow adipocytes than women of the same age [151,157].

On the other hand, evidence obtained recently from studies with rodents indicates that YAT is composed of two subtypes of adipocytes: constitutive yellow adipocytes (YAc) and regulatory yellow adipocytes (YAr) [148]. YAc are formed during the early stages of life, are maintained throughout the years in the appendicular skeleton (even in the presence of pathologies or environmental changes), and store mainly saturated lipids [148,149,150,157]. Likewise, YAc cells integrate a portion of bone marrow commonly called yellow due to its abundant large adipocytes [143,150]. As for YAr, their formation occurs in the axial skeleton within the red bone marrow, which gets its name from being composed primarily of hematopoietic tissue [148,156]. Unlike YAc, YAr accumulates mainly unsaturated lipids and tends to be smaller [148,149,156]. Likewise, YAr are more labile and respond to various physiological conditions [150,155,156]. For example, YAr and red bone marrow decrease with aging, obesity, hypertension, and T2DM, causing YAT to be composed predominantly of YAc [141,150,158]. This phenomenon is associated with reduced regenerative capacity and lower bone density, particularly in trabecular bone [149,151,155,157,158]. Additionally, recently published studies have suggested that the increase in adiposity percentage and waist circumference in humans is correlated with the risk of osteoporotic fractures, particularly in men and postmenopausal women [149,157,159]. Finally, these negative consequences on bone marrow health were also identified in people who abuse alcohol or in patients medicated with glucocorticoids, thiazolidinediones, or chemotherapy [148,149,155,157].

Knowledge about YAT in humans remains limited, as some limitations to studying these adipocytes are the difficulties in collecting and isolating these cells infiltrated in hematopoietic tissue [148,160]. For this reason, most studies are conducted in in vivo murine or in vitro human models [160]. Further studies are required to clarify the differences between YAT adipocyte subtypes (YAc/YAr), delve deeper into lipid accumulation or adipokine secretion mechanisms, and establish how YAT contributes to energy homeostasis [160]. Since its size can vary in response to increased fat intake, age, and visceral fat, it is also strongly influenced by estrogen, insulin, or glucocorticoid signals [148,160]. Finally, diseases such as anorexia nervosa and lipodystrophy are associated with decreased YAT [161,162].

### 3.7. Hepatic Stellate Cells (HSCs)—Blue Adipocytes

Hepatic stellate cells (HSCs) were first described in 1876 by Karl Kupffer and have been widely studied due to their critical roles in liver physiology and pathology [163,164]. In their quiescent state, these cells contain multiple lipid droplets measuring two to eight micrometers in diameter, an oval nucleus located centrally, and a developed Golgi apparatus and rough endoplasmic reticulum [163,164,165]. Furthermore, these cells have elongated processes in contact with endothelial cells [163].

HSCs, also referred to as blue adipocyte-like cells in some contexts, are located in the liver and positioned in the space of Disse, between sinusoidal endothelial cells and hepatocytes (Figure 3) [163,166,167]. They are also of mesenchymal origin and in humans are formed from the third month of gestation [163,164,166,168]. The importance of these adipocyte-like cells lies in the fact that they constitute approximately 10% to 15% of cells in the healthy liver and, due to their sensitivity to extracellular stimuli, they have very varied functions [167,168]. For example, intercellular communication, regulation of the remodeling of extracellular matrix components, regulation of sinusoidal blood flow, and communication with immune cells [166,169,170]. However, one of their best characterized functions in the quiescent state is the storage of lipid droplets containing triglycerides, cholesterol, and vitamin A in the form of retinol [165,166,169]. Retinol is the main component of these droplets, and 90% of hepatic vitamin A and 80% of the total is accumulated in them [163,166]. This characteristic is important because retinol regulates cell proliferation, differentiation, and morphogenesis [166,169]. In cell cultures, lipid droplets with vitamin A exhibit blue/green fluorescence at a wavelength of approximately 330 nm, giving them their characteristic name [165].

On the other hand, another vital function of HSCs is their ability to act as defense cells, responding to liver abnormalities such as infections, inflammation, tissue injury, or excess lipopolysaccharides [167,168,170]. During this process, HSCs become activated and interact with immune cells while promoting changes in the extracellular matrix [165,167]. During this process, these cells lose their lipid droplets, transdifferentiate into cells like myofibroblasts, and promote collagen production [165,166,167,168]. Likewise, these cells secrete pro-inflammatory cytokines, growth factors, leptin, and adiponectin [168]. These responses contribute to the removal of damaged hepatocytes and scar formation at the injury site, limiting further tissue damage; however, prolonged and repeated activation of this mechanism causes fibrosis, cirrhosis, and hepatocarcinogenesis [169,171,172].

In their quiescent state, HSCs, which share some adipocyte-like features, are not directly associated with liver or metabolic dysfunction [169]. Likewise, it does not appear to increase proportionately with pathologies such as obesity [166,169]. However, studies indicate that some triggers of stellate cell activation are obesity, dyslipidemia, hypertension, and T2DM [170]. These conditions may promote hepatic fibrosis through mechanisms in which inflammation plays a central role [171,172].

For this reason, further studies are required to clarify less explored roles of HSCs and their functional parallels with adipocytes. For example, their abundant expressions of leptin, adiponectin, PPAR-γ, and sterol regulatory element binding protein 1c (SREBP-1c) [164,173]. The latter two are characterized by being two key regulators of adipogenesis [174]. Once again, the main limitation to expanding our knowledge is the challenge of studying cells that are difficult to isolate in vivo [169]. However, the association between HSCs and adipocytes has been accentuated thanks to the studies by Tsukamoto and collaborators, which indicate that the transcriptional program involved in adipocyte differentiation overlaps with the program required to maintain HSCs in their quiescent vitamin A storing phenotype [164,173].

## 4. Anatomical Location of Adipose Tissue and Its Role in Obesity Progression

**Figure 4:**
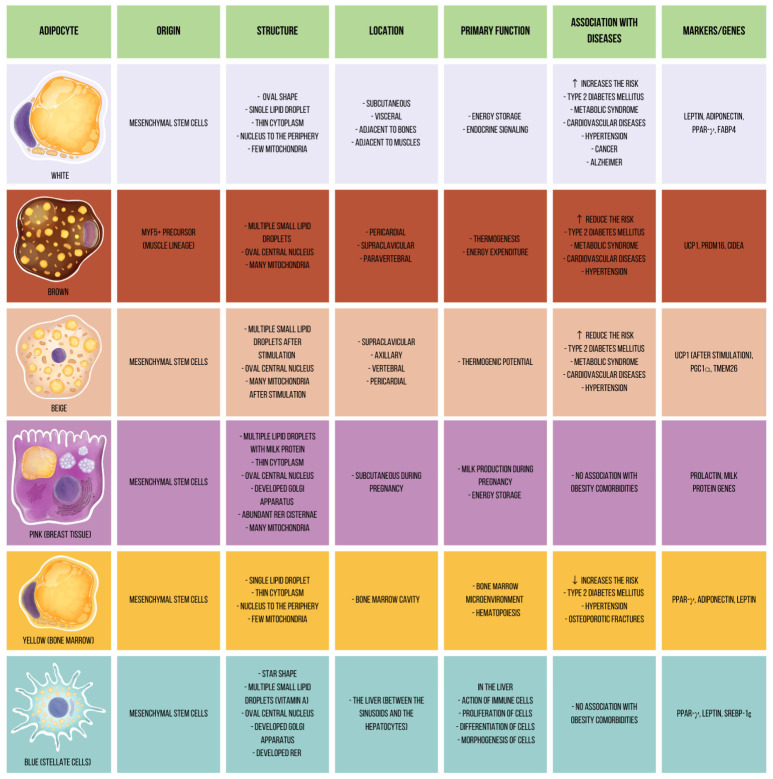
Morphology of Adipocytes. This figure summarizes the key attributes of different types of adipocytes, including their origin, structure, location, primary functions, and associations with metabolic or other health-related conditions. Adipocyte types included are white, brown, beige, pink (mammary tissue), yellow (bone marrow), and blue (stellate cells), highlighting their distinct roles and contributions to human physiology and disease. Upward and downward arrows indicate an increase or decrease, respectively, in the abundance or activity of the corresponding adipocyte population.

As previously mentioned, adipose tissue can be classified based on its physiology. The unique characteristics, molecular markers, origins, and physiological roles of the different types of adipose tissues, including WAT, BAT, BeAT, PAT, YAT, and BluAT (HSC), are summarized in Figure 4. However, this physiological classification should be complemented by anatomical location (Figure 3). Over the past 80 years, several studies have shown that adipocyte depot location influences lipid storage capacity, endocrine activity, lipolytic response, and sensitivity to physiological stimuli [175]. Consequently, adipose tissue distribution pattern may be more relevant than total adiposity percentage for the development of metabolic diseases [176,177].

**Figure 5:**
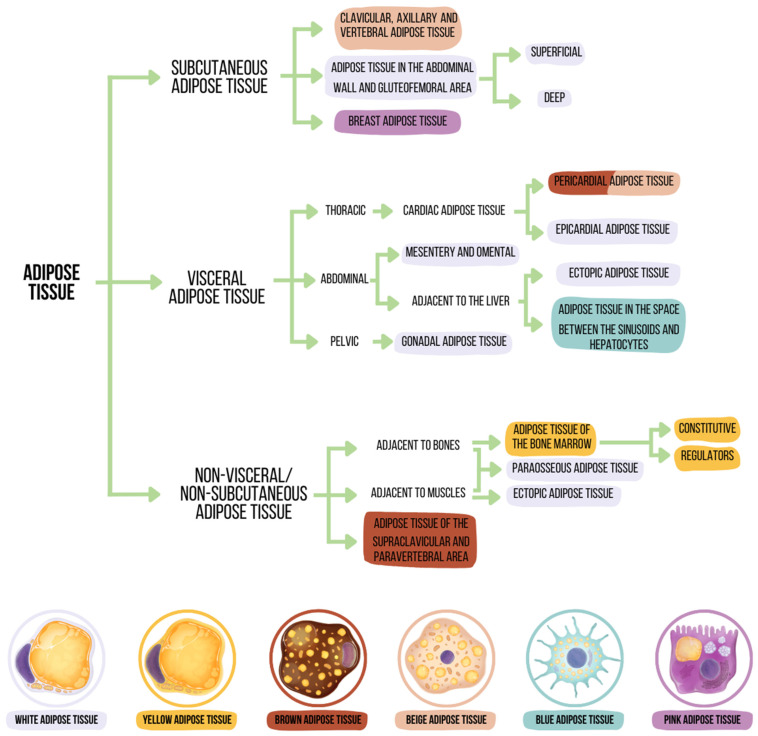
Classification of adipose tissue based on its physiology (white, brown, beige, pink, yellow, and blue) and anatomical location (subcutaneous, visceral, and non-visceral/subcutaneous adipose tissue).

This review suggests an integrative classification of adipose tissue that considers its physiology and anatomical location as factors in the analysis. It is important to emphasize that the anatomical distribution of adipose tissue is influenced by sex, age, diet, physical activity, and medication use. Based on studies conducted in healthy adults, adipose tissue can be anatomically classified into three main compartments: subcutaneous adipose tissue, visceral adipose tissue, and non-visceral/non-subcutaneous adipose tissue (Figure 5) [175,176].

### 4.1. Subcutaneous Adipose Tissue (SAT)

Subcutaneous adipose tissue (SAT) is located between the dermis and the connective membrane that covers the muscles (aponeurosis) [175]. SAT is generally composed of relatively small adipocytes that secrete abundant leptin, show high insulin sensitivity, and have a lower lipolytic rate than visceral adipose tissue (50% lower than visceral adipose tissue) [176,177] SAT is also highly dynamic, as specific stimuli can induce transdifferentiation toward pink adipocytes during pregnancy and lactation or beige adipocytes in response to cold exposure and exercise [117,121,136,139]. A recent single cell methylome and 3D genome atlas of human subcutaneous adipose tissue identified cell type-specific epigenomic programs and showed that abdominal obesity risk variants are preferentially enriched in adipocyte regulatory compartments, whereas inflammatory enrichment is concentrated in myeloid compartments. However, the most explored adipocytes of the SAT are the white ones, since they are more abundant (80% of the total fatty tissue) (Figure 5) [176]. However, excessive expansion of white adipocytes within SAT may contribute to obesity-related diseases [177].

Regarding its location, SAT is found in the clavicular, axillary, and vertebral regions (beige subcutaneous adipose tissue), abdominal wall, breast tissue (pink subcutaneous adipose tissue), and gluteofemoral area (Figure 5) [169,170,171]. This last depot is generally the largest because it constitutes 50% of the total SAT and, due to its abundance of estrogen receptors, accumulates more in women than in men [176,177,178].

It should be noted that white SAT expansion is significantly influenced by hormonal changes, particularly those related to sex hormones [179]. For example, estrogen increases the size and number of subcutaneous adipocytes and attenuates lipolysis [179,180]. Consequently, preferential accumulation of SAT (particularly in the gluteofemoral region) is promoted [180]. At the same time, androgen levels are inversely correlated with white SAT deposition, contributing to its lower abundance in men [179].

On the other hand, the white SAT stores large amounts of lipids, expanding primarily through hyperplasia (an increase in the number of adipocytes) [176]. For this reason, white SAT is considered a metabolic buffer that can temporarily limit the adverse effects of energy surplus and is associated with a lower risk of metabolic diseases [176,177,180]. However, despite the protective effect of white SAT, when the tissue’s storage capacity is exceeded, the mechanism for generating new adipocytes deteriorates [177]. Consequently, inflammation, insulin resistance, and ectopic lipid accumulation (skeletal muscle, heart, and liver) are promoted [176,177,178]. For this reason, excess white SAT over the long term is associated with elevated blood levels of triglycerides, cholesterol, and glucose [178]. Likewise, studies reveal a relationship between high percentages of white SAT and hypertension, heart disease, and T2DM [176,178]. In this context, lipid storage in white SAT may delay metabolic deterioration, although it does not fully prevent it [176,177,178,180].

Hormonal stimulation during puberty, pregnancy, and menopause may promote pathological growth of subcutaneous adipose tissue [179]. For example, loss of ovarian function increases the risk of diseases such as metabolic syndrome and T2DM, which increases cardiovascular risk [177,179]. Furthermore, menopause is associated with redistribution and morphological changes in the white SAT [179]. This remodeling is characterized by adipocyte hypertrophy, fibrosis, and chronic inflammation, which may reinforce insulin resistance and perpetuate tissue dysfunction [171,179].

On the other hand, the participation of various hormones, such as cortisol, can influence the expansion of this tissue, since cortisol is generated in the white SAT by the enzyme 11β-hydroxysteroid dehydrogenase type 1 (11βHSD1), which converts inactive cortisone into active cortisol [181]. In consequence, the activity of this enzyme in this tissue increases body weight [179,181]. Likewise, growth hormone (GH) can modify the white SAT by affecting its mass, distribution, composition, endocrine function, and inflammatory and fibrotic status. Previous studies indicate that humans and rodents with elevated GH levels experience a decrease in longevity and are more susceptible to cardiovascular diseases and cancer [182]. Finally, insulin plays a central role in the expansion of white SAT by stimulating lipogenesis and inhibiting lipolysis, favoring fat storage in conditions of energy surplus [178].

The deep fascia divides white SAT into two compartments: superficial SAT, which protects organs from external compression, and deep SAT, which contributes to the mechanical adaptation of the skin in response to external forces [176,178,183]. Superficial white SAT is composed of small, organized adipocytes that form a thin layer of compact tissue close to the skin of the abdomen [178,183,184]. It also predominantly accumulates monounsaturated fatty acids and secretes abundant leptin and adiponectin [185,186]. In contrast, deep SAT has irregularly distributed adipocytes that form a thick layer of loose, highly vascularized tissue adjacent to muscle fascia [184]. For this reason, deep SAT is a major site of adiposity accumulation [184,185,186].

Previous studies indicate that superficial and deep SAT differ in characteristics that may influence their associations with obesity-related diseases [183,186]. For example, deep SAT exhibits greater lipolytic activity, greater infiltration of pro-inflammatory cells, and lower adiponectin secretion compared to superficial SAT [187,188]. Additionally, deep SAT shows elevated gene expression levels of leptin, lipoprotein lipase, fatty acid synthase (FASN), IL-6, monocyte chemoattractant protein 1 (MCP-1), and TNF-α. As a result, deep SAT has a diminished capacity for lipid storage and a tendency toward chronic inflammation [187,188,189].

For this reason, deep SAT has been suggested to expand excessively in individuals with obesity, with features that partially resemble visceral adipose tissue [185,186]. Likewise, it is proposed that its increase is associated with altered systemic metabolism, especially in men [190,191,192]. This could be partially explained by the fact that in men, the increase in subcutaneous adiposity occurs mainly in the deep SAT. On the contrary, in women, both the deep and superficial SAT expand proportionally [190].

Deep SAT appears to be associated with several obesity-related pathologies in both sexes. For example, T2DM, metabolic syndrome, non-alcoholic fatty liver disease, hypertension, and heart disease [184,190]. As measures to reduce SAT (deep and superficial SAT), physical activity and dietary changes are recommended [192,193]. Likewise, surgical procedures such as liposuction and lipectomy are also very effective (mainly for decreasing deep SAT). However, some questions have been raised about the safety of these procedures [193,194,195]. For example, considering the initial protective effect of superficial SAT, is it possible that the reduction of this tissue generates adverse metabolic effects? [194]. Likewise, because of the adipose tissue regulation system, could the sudden elimination of the white SAT favor activating recovery mechanisms for the removed tissue? [195]. Studies in rodents fed a high-fat diet and subsequently undergoing lipectomy observed insulin resistance and increased triglyceride concentrations [194]. Furthermore, weeks after the surgical intervention, they quantified an increase in adipose tissue in areas of white SAT not subjected to lipectomy [195]. However, it is essential to note that studies conducted with humans do not generate sufficient evidence to establish benefits or contraindications [177,185,194].

Another adipose compartment adjacent to SAT, known as dermal white adipose tissue (DWAT), has recently received increasing attention. DWAT, as its name suggests, is a layer of white adipocytes located within the reticular dermis of the skin [188,189,196]. Specifically, in rodents, this layer is 2 to 15 cells thick and is found in the panniculus carnosus between the skin and the deep fascia of the DWAT [187,188]. In humans, these adipocytes are concentrated in a conical arrangement around the shaft units, hair follicles, and the sebaceous glands of the cheek, neck, chest, abdomen, and buttocks [187,196].

Another notable difference from the SAT is that, due to its transdifferentiation capacity, DWAT has functions not directly associated with metabolic homeostasis, as it participates in the hair cycle, wound healing, thermogenesis, and immune defense against infections [187,188]. After skin injury, mature adipocytes in DWAT can undergo lipolysis and dedifferentiate into fibroblast-like cells [196]. Furthermore, these dermal adipocytes are rich in adipokines, and cytokines are released in response to injury [188]. For this reason, DWAT is a relevant area of research due to its plasticity and role in immune defense [187,196].

### 4.2. Non-Visceral/Non-Subcutaneous Adipose Tissue

Non-visceral or non-subcutaneous adipose tissue is found in several anatomical regions, including the supraclavicular and paravertebral areas (composed of brown adipocytes), within bone marrow, where yellow adipocytes predominate; within skeletal muscle, where intramuscular white adipocytes are present; and at the muscle–bone interface, where paraosseous white adipocytes can be found (Figure 5) [185].

The accumulation of non-visceral or non-subcutaneous adipose tissue composed of white adipocytes is favored by aging, obesity, and physical inactivity [176,177]. In addition, it decreases with physical activity [176]. These compartments are challenging to study in humans because they are difficult to dissect and generally require high-resolution imaging methods for visualization [169,170]. However, rodent studies suggest that internal non-visceral white adipose tissue accumulation may adversely affect metabolic health, as this tissue is associated with inflammatory processes and insulin resistance [175,176,177].

### 4.3. Visceral Adipose Tissue (VAT)

Visceral adipose tissue (VAT) covers the internal organs (white adipose tissue and, to a lesser extent, brown and beige adipose tissue). In addition, it is also found within them (blue adipose tissue) as seen in Figure 5. The VAT constitutes five to eight percent of total adipose tissue in women and 10 to 20% in men [189].

In white VAT, adipocytes are generally larger, and their secretory profile is predominantly proinflammatory, with increased secretion of IL-6, TNF-α, and resistin [176,178]. Additionally, unlike white SAT, white VAT secretes less adiponectin, is highly vascularized, shows abundant infiltration of proinflammatory cells, has higher lipolytic activity, and expands predominantly through hypertrophy (an increase in adipocyte size) [11,177,178].

These characteristics make white VAT a less suitable depot for long-term lipid storage [197]. Moreover, this tissue significantly promotes inflammation, hyperglycemia, dyslipidemia, and insulin resistance [176,178]. Consequently, epidemiological studies indicate a higher risk of developing T2DM, MAFLD, metabolic syndrome, cardiovascular diseases, hypertension, certain types of cancer (colon, breast, and prostate), and stroke [11,176,177,178,182]. These risks associated with white VAT accumulation are clinically relevant, and, due to the large number of androgen receptors in this tissue, accumulation is greater in men than in women [176,177]. However, it should be noted that the percentage of visceral adiposity increases in women with menopause [176].

It should be emphasized that some studies using animal models have indicated that not all white VAT is the same. Therefore, it has been divided into three main anatomical compartments: thoracic visceral adipose tissue (adjacent to the heart), abdominal visceral adipose tissue (retroperitoneal and intraperitoneal), and pelvic visceral adipose tissue (gonadal) [175,177]. However, the applicability of this classification to humans remains under investigation [177].

Thoracic visceral adipose tissue, one of the most studied visceral depots because of its physiological relevance, includes epicardial adipose tissue located between the visceral pericardium and the outer surface of the myocardium [175,198]. Under physiological conditions, epicardial adipose tissue represents approximately 20% of the heart mass and is commonly found in the atrioventricular and interventricular grooves [198]. This tissue serves as a local energy source and is composed of white adipocytes [175]. However, excessive accumulation of this tissue may impair cardiac function by increasing mechanical load during cardiac contraction [198]. In addition, the adipose tissue pericardial is a source of pro-inflammatory cytokines associated with adverse cardiovascular conditions and coronary artery calcifications [199]. Likewise, thoracic adipose tissue has been associated with pulmonary hypertension and may exert local and systemic effects [199,200,201]. Also, adipose tissue adjacent to the lungs, together with its secreted proinflammatory factors, may indirectly contribute to the severity of influenza virus infection [200,201].

In the case of abdominal visceral adipose tissue, it drains into the portal vein and has its main sites of accumulation in the mesentery and omentum [175,198]. Likewise, as in thoracic adipose tissue, the abdominal visceral adipose tissue is composed of white adipocytes [177].

Its expansion is a hallmark of central obesity and is strongly associated with insulin resistance, thrombotic vascular disease, kidney damage, and local intestinal inflammation, which may contribute to conditions such as Crohn’s disease (CD) and mucosal ulceration [177,198,202,203,204]. It should be noted that the mechanisms underlying pathologies associated with mesenteric adipose tissue are only partially understood [202]. However, it is well known that visceral obesity promotes infiltration of macrophages, the translocation of bacteria from the intestinal lumen (resulting in creeping fat formation surrounding the inflamed bowel), and the secretion of pro-inflammatory adipokines and cytokines, including TNF-α, IL-1, IL-6, and IL-8 [202,203].

Another consequence of the excessive increase of abdominal visceral adipose tissue is hypertrophied adipocytes [177,200]. This may contribute to increased intra-abdominal pressure in individuals with obesity, while hypertrophic adipocytes may become more susceptible to rupture under ordinary physical forces (cough and/or exercise) [177]. In addition, adipose cell apoptosis starts a cascade, leading to a chronic inflammatory state. For example, cytokine release may stimulate hepatic C-reactive protein (CRP) production, while immune cells accumulate around ruptured adipocytes and residual lipid droplets, and cells sequester residual lipid droplets [177,200].

Finally, pelvic visceral adipose tissue is in the periphery of the gonads (bladder, uterus/testes, rectum) and is formed of white adipocytes [175]. Studies in rodents indicate that this type of tissue grows primarily in the initial phase of weight gain and is the first region of visceral adipose tissue to increase [196,197]. Moreover, they concluded that upon reaching its maximum storage capacity, pelvic visceral adipose tissue promotes the accumulation of abdominal visceral adipose tissue [205,206].

Some consequences of the exacerbated presence of pelvic visceral adipose tissue are increased infiltration of pro-inflammatory macrophages and insulin resistance [206]. In addition, this adipose tissue has been proposed as a marker of oncologic spread and has been associated with endometriosis, in which fibrotic adipose tissue may be found adjacent to the peritoneal region [207,208]. Finally, excessive deposition of mature adipose tissue in the pelvic cavity is characteristic of pelvic lipomatosis, a disease characterized by the deposition of mature fat tissue in the pelvic cavity without delimitation by a capsule [206,209]. This results in inflammation and compression of the bladder, rectum, and blood vessels. Its incidence remains undetermined but is most prevalent in men and dark-skinned individuals [209].

Unlike the white SAT, liposuction is not a viable or applicable strategy to decrease the percentage of white VAT. White VAT is mainly reduced through lifestyle modification and, when indicated, pharmacotherapy [175,189]. Further studies on this adipose compartment are needed to better understand its contribution to the progression of obesity-related diseases.

### 4.4. Perivascular Adipose Tissue (PVAT)

Perivascular adipose tissue (PVAT) surrounds most blood vessels, with the notable exception of pulmonary and cerebral vasculature, and was historically regarded as inert structural support. PVAT is now recognized as an active paracrine organ with direct outside-in signaling to the vessel wall. Depending on vascular bed, PVAT can resemble either brown or white adipose tissue; thoracic (periaortic) PVAT shares thermogenic, UCP-1-expressing characteristics with BAT, while PVAT surrounding other vessels is more white-adipocyte-like, and recent evidence suggests PVAT arises from its own dedicated precursor population distinct from adjacent subcutaneous or visceral depots.

In the healthy state, PVAT exerts anticontractile and anti-inflammatory effects on the vessel wall through secretion of adiponectin, nitric oxide, and omentin, helping maintain vascular tone and endothelial function [210]. In obesity, hypertension, and atherosclerosis, PVAT undergoes phenotypic remodeling, including reduced adiponectin secretion, macrophage infiltration, and a shift toward a pro-inflammatory, pro-oxidative secretory profile, that promotes vascular inflammation and contributes to disease progression. Clinically, non-invasive CT-derived indices of PVAT attenuation are being explored as imaging biomarkers of vascular inflammation, and reductions in these indices have been observed following statin therapy and anti-inflammatory treatment, highlighting PVAT’s potential as both a biomarker and therapeutic target in cardiovascular disease [211].

However, whether PVAT dysfunction is a cause or consequence of vascular disease in humans, and whether PVAT-targeted therapies (as opposed to biomarker use) are feasible, remain open questions.

## 5. Conclusions

The study of adipose tissue has provided valuable insights, revealing that different adipose tissue depots possess distinct and previously underestimated functions. These findings underscore that the predisposition to dysfunction varies significantly across different fat distribution areas, as they exhibit varying levels of hypoxia, adipokine secretion alterations, and immune cell infiltration. Consequently, the risk of developing conditions such as T2DM, hypertension, and cardiovascular disease increases, with the loss of adipose tissue diversity (decrease in brown, beige, pink, yellow, and blue adipose tissue) and with the increase in white adipose tissue (especially in the visceral area).

Analyzing adipose tissue from physiological and anatomical perspectives is essential to comprehending the mechanisms underlying the progression of obesity-related pathologies and identifying potential therapeutic approaches. Moreover, recent advances highlight the possible roles of lesser-studied adipose tissue types (beige and brown adipose tissue) in metabolic regulation and disease prevention, offering promising areas for targeted intervention.

Despite this progress, significant gaps remain in our understanding. Across the adipocyte populations discussed in this review, several open questions recur: the human relevance of rodent-derived lineage and macrophage-polarization models, the relative contribution of distinct insulin-resistance pathways in human tissue, the translational ceiling of BAT-directed therapy given limited adult BAT mass and interindividual variability, and the causal versus consequential role of depot-specific dysfunction (e.g., in perivascular adipose tissue) in disease progression. Addressing these gaps will likely require greater reliance on human single-cell and spatial transcriptomic data, standardized in vitro models of human adipocyte subtypes, and longitudinal human studies linking depot-specific remodeling to clinical outcomes. Expanding research in these areas may contribute to the development of more precise approaches to address the complexity of obesity and its associated disorders.

## Data Availability

No new data were created or analyzed in this study. Data sharing is not applicable to this article.

## Abbreviations

The following abbreviations are used in this manuscript:

11βHSD1	11β-hydroxysteroid dehydrogenase type 1
18 FDG-PET/CT	Positron emission tomography with fluorine-18-fluorodeoxyglucose
BAT	Brown adipose tissue
BeAT	Brite adipose tissue
BMI	Body mass index
CD	Crohn’s disease
CRP	C-reactive protein
DHA	Docosahexaenoic acid
DWAT	Dermal white adipose tissue
EPA	Eicosapentaenoic acid
FASN	Fatty acid synthase
FCs	Futile cycles
FFAs	Free fatty acids
FGF-1	Fibroblast growth factor 1
GH	Growth hormone
GLUT-4	Glucose transporter 4
HSCs	Hepatic stellate cells
IGF-1	Insulin-like growth factor 1
IR	Insulin resistance
IRS-1	Insulin receptor substrate 1
MAFLD	Metabolic-associated fatty liver disease
MAPK	Mitogen-activated protein kinase
MC4R	Melanocortin-4 receptor
MCP-1	Monocyte chemoattractant protein 1
NCDs	Non-communicable diseases
NF-κB	Nuclear factor kappa B
PAT	Pink adipose tissue
PI3K	Phosphatidylinositol-3-kinase
PPAR-γ	Peroxisome proliferator-activated receptor gamma
SAT	Subcutaneous adipose tissue
SPP1	Secreted phosphoprotein 1
SREBP-1c	Sterol regulatory element binding protein 1c
SVF	Stromal vascular fraction
T2DM	Type 2 diabetes mellitus
TNF-α	Tumor necrosis factor-alpha
UCP-1	Uncoupling protein 1
VAT	Visceral adipose tissue
WAP	Whey acidic protein
WAT	White adipose tissue
WHO	World Health Organization
YAc	Constitutive yellow adipocytes
Yar	Regulatory yellow adipocytes
β3-AR	β3-adrenergic receptor
ω-3 PUFA	Omega-3 polyunsaturated fatty acids
